# Transmission of *Acinetobacter baumannii* by Surgical Masks During the COVID-19 Pandemic

**DOI:** 10.1017/ash.2021.97

**Published:** 2021-07-29

**Authors:** Lisa Saidel, Abraham Borer, Orli Sagi

## Abstract

**Background:** *Acinetobacter baumannii*, one of the major causes of nosocomial infections in modern healthcare systems, is characterized by its great persistence in the environment and by its ability to rapidly develop resistance to many antimicrobials. Most *A. baumannii* infections occur in intubated critically ill patients, causing ventilator-associated pneumonia which is a leading cause of mortality. During the coronavirus disease 19 (COVID-19) pandemic an increase in hospital-acquired carbapenem-resistant *A. baumannii* (CRAB) infection and colonization in acute-care hospitals has been described. CRAB healthcare-associated infections are often linked to breaches of infection prevention and control (IPC). Beginning in April 2020, our hospital’s IPC unit ordered mandatory universal masking for all healthcare workers (HCWs). Shortages of personal protective equipment during the COVID-19 pandemic led to extended use of surgical face masks by HCWs in our hospital. We investigated whether the extended use of surgical face masks was linked to an increase of CRAB colonization in our intubated critically ill patients. **Methods:** Surgical masks were collected from doctors, nurses, and housekeeping staff working in 2 internal medicine departments, each including a 4-bed unit for intubated critically ill patients. All surgical masks were worn continuously for 4–5 hours before removal. “Cases“ were defined as HCWs who treated CRAB colonized critically ill patients. “Controls“ were defined as HCWs who did not enter the critically ill patient unit. Surgical masks were incubated with BHI enrichment broth (HyLabs Rehovot, Israel) for 48 hours at 35°C. BHI was seeded on multidrug-resistant (MDR)–selective CHROMagar plates (HyLabs) and incubated overnight at 35°C. Identification was performed using MALDI-ToF mass spectrophotometry (bioMérieux, France). Susceptibility was tested using Vitek 2 (bioMérieux). **Results:** In total, 55 HCWs participated in the study: 25 cases and 30 controls. Masks from 10 cases (40%) were colonized with *Acinetobacter* spp versus only 3 masks (10%) from controls (OR, 5.98; 95% CI, 1.42–25; *P* = .012). Of 13 masks contaminated with *Acinetobacter* spp, 8 of 10 contaminated masks among cases were colonized with CRAB, whereas only 1 of 3 masks of controls was colonized with CRAB. **Conclusions:** During the COVID-19 pandemic, extended surgical mask use while treating patients colonized with CRAB increased mask contamination with this bacterium. Surgical masks should be changed after treating a patient colonized with CRAB the same way gown and glove removal and hand hygiene are performed.

**Funding:** No

**Disclosures:** None

